# Two novel Co(II) complexes with two different Schiff bases: inhibiting growth of human skin cancer cells

**DOI:** 10.1590/1414-431X20176390

**Published:** 2017-07-03

**Authors:** Y.-J. Xiao, Q.-C. Diao, Y.-H. Liang, K. Zeng

**Affiliations:** 1 Department of Dermatology, Nanfang Hospital, Southern Medical University, Guangzhou, Guangdong, China; 2 Department of Dermatology, The Chongqing Hospital of Traditional Chinese Medicine (The First People's Hospital of Chongqing City), Chongqing, China; 3 Department of Dermatology, Shenzhen Hospital, Southern Medical University, Shenzhen, Guangdong, China

**Keywords:** Schiff bases, Coordination compound, Antitumor activity

## Abstract

Using two flexible Schiff bases, H_2_L_1_ and H_2_L_2_, two new cobalt II (Co(II))-coordination compounds, namely, Py_3_CoL_1_ (1) and Py_3_CoL_2_ (2) (Py=pyridine, L_1_=3,5-ClC_6_H_2_(O)C=NC_6_H_3_(O)-4-NO_2_, L_2_=3,5-BrC_6_H_2_(O)C=NC_6_H_3_(O)-4-NO_2_) have been synthesized under solvothermal conditions. Single crystal X-ray structural analysis revealed that compounds 1 and 2 are both six-coordinate in a distorted octahedral geometry, and the 1D chain structure was formed by the π…π and C-H…O interactions or C-H…Cl interaction. The *in vitro* antitumor activities of 1, 2 and their corresponding organic ligands Py, L_1_, and L_2_ were studied and evaluated, in which three human skin cancer cell lines (A-431, HT-144 and SK-MEL-30) were used in the screening tests.

## Introduction

Cancer is a proliferation disorder disease with apoptosis obstacles (1,2). It strikes more than one-third of the world's population and causes over 20% of all deaths (3). Standard cancer treatment protocols include surgery, radiotherapy and chemotherapy (4). Unfortunately, chemotherapy is not effective in treating cancers associated with innate resistance to apoptosis and/or acquired resistance to drugs during treatment. Discovery of novel effective anticancer medicines is therefore of great importance (5).

**Figure 1.**
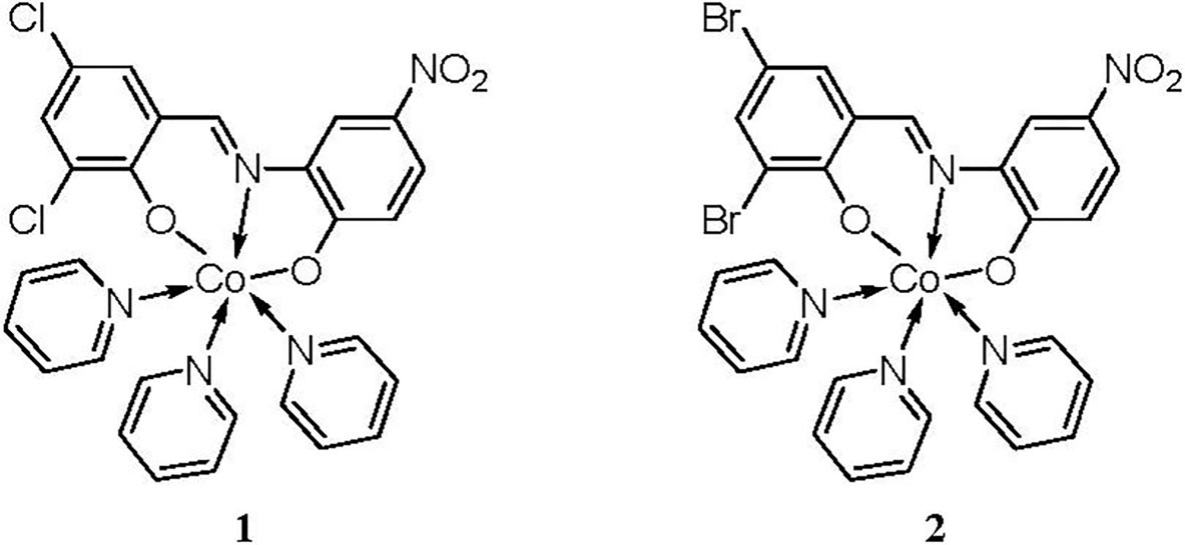
Scheme representation of compounds Py_3_CoL_1_ (1) and Py_3_CoL_2_ (2).

Cobalt complexes with Schiff bases have received considerable attention in the fields of coordination chemistry and biological chemistry (6). Cobalt functions as the active site of hydrolytic enzymes, such as carboxypeptidase and carbonic anhydrase where it is in a hard-donor coordination environment of nitrogen and oxygen (7). Cobalt has been recognized as an important cofactor in biological molecules, either as a structural template in protein folding or as a Lewis acid catalyst that can readily adopt four-, five-, or six-coordination (7,8). The cobalt (Co) complexes with the Schiff bases derived from salicylaldehyde and its analogues have been extensively studied (9). In this work, two new Co(II) complexes, Py_3_CoL_1_ (1) and Py_3_CoL_2_ (2) (Figure 1) (Py=pyridine, L_1_=3,5-ClC_6_H_2_(O)C=NC_6_H_3_(O)-4-NO_2_, L_2_=3,5-BrC_6_H_2_(O)C=NC_6_H_3_(O)-4-NO_2_), were solvothermally prepared by employment of two different base ligands and their antitumor activities were then evaluated.

## Material and Methods

### Apparatus and materials

All starting materials and reagents used in this research were obtained commercially and used without further purification. Element analyses (C, H, and N) were determined with an elemental Vario EL III analyzer (Elementar, Germany). Single-crystal X-ray diffraction data for compounds 1 and 2 were recorded on Mercury CCD diffractometer (Bruker Optics, Germany). The melting points were taken on a XT-4 micro melting apparatus (Ledon, China), and the thermometer was uncorrected. Three human skin cancer cell lines (A-431, HT-144 and SK-MEL-30) were purchased from Sigma-Aldrich (USA).

### Synthesis and characterization of compounds 1 and 2

A mixture of CoCl_2_ (1.0 mmol, 0.130 g), and 3,5-dichlorosalicylaldehyde-2-amino-4-nitrophenol Shiff base (H_2_L_1_, 1.0 mmol, 0.327 g) were mixed in 25 mL methanol. After heating and dissolving in air, 10 mL methanol solution of CoCl_2_ (50 mM) was added to the reaction flask. Most of the solvent was removed by rotary evaporators after 2 h of reaction. Then, pyridine was dropped to dissolve, and the solution continued to reflux for 2 h. The solution was cooled down to room temperature and filtered and the brown crystals of solution 1 were obtained. Analytical characteristics found for compound 1 (C_28_H_21_Cl_2_CoN_5_O_4_) were: C, 54.18; H, 3.39; N, 11.30%. Calculate: C, 54.13; H, 3.41; N, 11.27%.

The synthesis method for compound 2 was similar to that of compound 1. Analytical characteristics found for compound 2 (C_28_H_21_Br_2_CoN_5_O_4_) were: C, 47.40; H, 3.00; N, 9.85%. Calculate: C, 47.35; H, 2.98; N, 9.86%.

### Crystal structure determination

Structural measurement was performed on the computer-controlled Mercury CCD diffractometer with graphite-monochromated Mo-*Kα* radiation (*λ=*0.71073 Å) at *T=*293 (2) K. Absorption correction was made using the SADABS (Bruker AXS Inc., USA) program. The structure was solved using the direct method and refined by full-matrix least-squares methods on *F* ^2^ using the SHELXS-97 program package (10). Crystallographic data and structural refinements for compounds 1 and 2 are summarized in Table 1.

**Table 1.** Crystal data and structure refinement for Py_3_CoL_1_ (1) and Py_3_CoL_2_ (2).

	1	2
Formula	C_28_H_21_Cl_2_CoN_5_O_4_	C_28_H_21_Br_2_CoN_5_O_4_
*M*r	621.33	710.25
Temperature/K	296 (2)	296 (2)
Crystal system	Triclinic	Triclinic
Space group	*P*ī	*P*ī
*a*/Å	8.8780 (13)	8.8470 (7)
*b*/Å	9.0856 (13)	9.1799 (8)
*c*/Å	17.703 (3)	18.0141 (15)
*α*/°	104.445 (2)	104.335 (1)
*β*/°	92.184 (2)	91.368 (1)
*γ*/°	99.919 (2)	99.064 (1)
*V*/Å^3^	1357.2 (3)	1396.7 (2)
*Z*	2	2
*D*_calc_/g·cm^-3^	1.520	1.689
*μ*(Mo Kα)/mm^-1^	0.874	3.519
*θ* range/°	2.35 to 28.03	2.32 to 25.99
Reflections collected	17,238	15,294
No. unique data [*R*(int)]	6459 [0.0262]	5441 [0.0232]
No. data with *I* ≥ 2*σ*(*I*)	4,846	4,122
*R*_1_	0.0391	0.0328
*ωR*_2_(all data)	0.1058	0.0836
CCDC	960,791	960,795

CCDC numbers for compounds 1 and 2 contain the supplementary crystallographic data for this paper. These data can be obtained free of charge via http://www.ccdc.cam.ac.uk/conts/retrieving.html (or from the CCDC, 12 Union Road, Cambridge CB2 1EZ, UK; Fax: +44-1223-336033; E-mail: deposit@ccdc.cam.ac.uk)

### Antitumor activity

Stock solutions of 1, 2 and their corresponding organic ligands Py, L_1_ and L_2_ were prepared in DMSO and kept at -20°C. Appropriate dilutions of the compounds were freshly prepared just prior to the assays. Final concentrations of DMSO did not interfere with the cell growth.

Three human skin cancer cell lines (A-431, HT-144 and SK-MEL-30) grown as monolayer were routinely maintained in RPMI-1640 medium supplemented with 5% heat inactivated FBS, 2 mM glutamine and antibiotics (penicillin 100 U/mL, streptomycin 100 μg/mL), at 37°C in a humidified atmosphere containing 5% CO_2_. Exponentially growing cells were obtained by plating 1.5×10^5^ cells/mL for A-431 and HT-144 and 0.75×10^4^ cells/mL for SK-MEL-30, followed by 24 h of incubation. The effect of the vehicle solvent (DMSO) on the growth of these cell lines was evaluated in all the experiments by exposing untreated control cells to the maximum concentration (0.5%) of DMSO used in each assay.

## Results and Discussion

### Molecular structure

The crystal structure determined by single-crystal X-ray diffraction showed that 1 and 2 both crystallize in the triclinic system, space group *P*ī. The asymmetric unit comprises one Co(II) atom, three pyridine molecules, one 3,5-dichlorosalicylaldehyde-2-amino-4-nitrophenol or 3,5-dibromosalicylaldehyde-2-amino-4-nitrophenol Shiff base, respectively.

**Figure 2.**
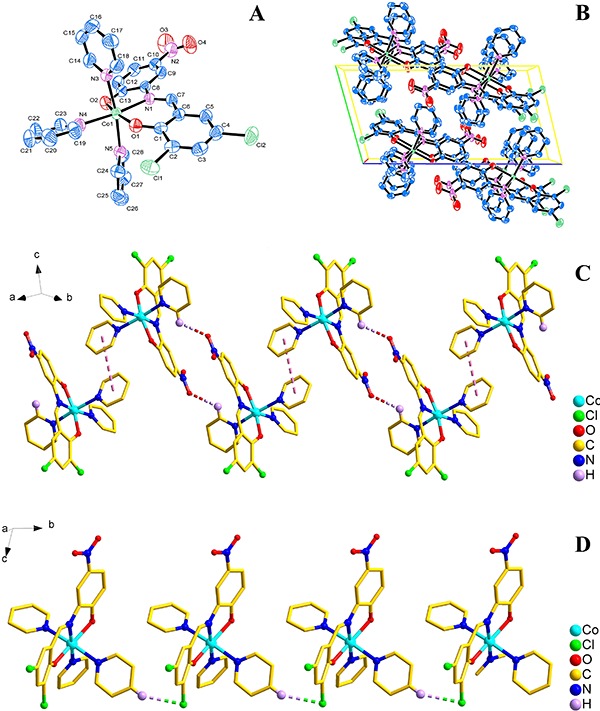
*A*, Molecular structure of compound 1 (Py_3_CoL_1_); *B*, packing of compound 1 in unit cell; *C*, 1D infinite chain structure of compound 1 was formed by the π…π and C-H…O interactions; *D*, 1D infinite chain structure of compound 1 was formed by the C-H…Cl interactions.

**Figure 3.**
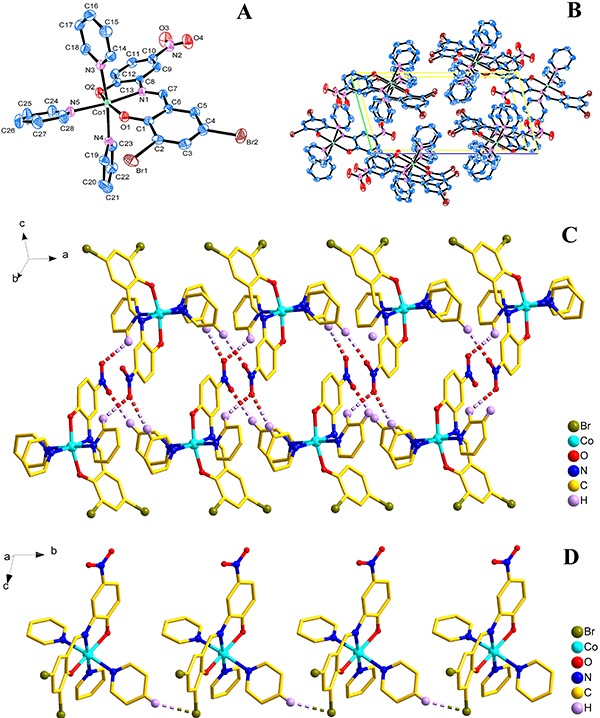
*A*, Molecular structure of compound 2 (Py_3_CoL_2_); *B*, packing of compound 2 in unit cell; *C*, 1D ribbon-like structure of compound 2 was formed by the C-H…O hydrogen bonding interaction, *D*, 1D chain-like structure of compound 2 was formed by the C-H…Br interaction.

As shown in Figures 2A and 3A, the central Co1 atom is six-coordinate in a distorted octahedral geometry and is surrounded by two oxygen atoms (O1 and O2) and one amino nitrogen atom (N1) from the ligand, and three nitrogen atoms (N3, N4, and N5) from three different pyridines. The axes positions were occupied by two nitrogen atoms (N1 and N4 for 1; N1 and N5 for 2) from Shiff base ligand (3,5-dichlorosalicylaldehyde-2-amino-4-nitrophenol for 1; 3,5-dibromosalicylaldehyde-2-amino-4-nitrophenol for 2) and one pyridine molecule, respectively. The angle of N1-Co1-N4 and N1-Co1-N5 is 173.94° and 173.92°, respectively, which obviously deviates from linear angle 180°. The equatorial positions were occupied by four atoms (O1, O2, N3 and N5 for 1; O1, O2, N3 and N4 for 2). The bond lengths and bond angles between the four atoms in the equatorial plane and the center of the Co1 atom are also different [for 1, Co1-O1=2.0116 (14) Å, Co1-O2=2.0609 (15) Å, Co1-N3=2.2210 (18) Å, Co1-N5=2.2029 (18) Å, ∠O1-Co1-N3=93.85 (6)°, ∠O2-Co1-N3=92.48 (7)°, ∠O1-Co1-N5=86.86 (6)°, ∠O2-Co1-N5=88.57 (7)°; for 2, Co1-O1=2.0157 (19) Å, Co1-O2=2.0610 (19) Å, Co1-N3=2.217 (2) Å, Co1-N4=2.207 (2) Å, ∠O1-Co1-N3=93.81 (8)°, ∠O2-Co1-N3=92.92 (9)°, ∠O1-Co1-N4=86.66 (8)°, ∠O2-Co1-N4=88.39 (9)°], so the central Co1 atom is six-coordinate in a distorted octahedral geometry in the complexes 1 and 2.

The packing of the compounds 1 and 2 in unit cell is shown in Figures 2B and 3B, respectively. Moreover, for 1, the π…π and C-H…O interactions were observed between adjacent molecules, which led to the formation of an interesting 1D chain structure (Figure 2C). The C-H…Cl interaction in an adjacent molecule also resulted in the formation of a 1D chain structure (Figure 2D); for 2, the C-H…O Hydrogen bonding interactions [H17…O4^i^ 2.5939 (29) Å, ∠C17-H17…O4^i^ 171.995 (232)°, i: 1+x, y, z; H25…O3^ii^ 2.5571 (30) Å, ∠C25-H25…O3^ii^ 136.210 (306)°, ii: 2-x, 2-y, -z; H23…O3^iii^ 2.5816 (35) Å, ∠C23-H23…O3^iii^ 129.866 (196)°, iii: 1-x, 2-y, -z;] were observed between adjacent molecules, which led to the formation of an interesting 1D ribbon-like structure (Figure 3C). The C-H…Br interaction [H21…Br1^i^ 2.8754 (4) Å, ∠C21-H21…Br1^i^ 149.139 (227)^o^, i: x, 1+y, z] in an adjacent molecule also resulted in the formation of a 1D chain-like structure (Figure 3D).

### Antitumor activity

The tumor cell growth inhibition activities of 1, 2 and their corresponding organic ligands Py, L_1_ and L_2_ were assessed *in vitro* on 3 human skin cancer cell lines (A-431, HT-144, and SK-MEL-30) after continuous exposure for 48 h. The results were compared to the antiproliferative effects of the reference control doxorubicin. All compounds were dissolved in DMSO at 1 mg/mL immediately before use and diluted just before addition to the cell culture.

Data are reported as means±SE of 3 independent experiments performed in duplicate (Table 2). The antiproliferative activity of the test compounds against each of the title tumor cell lines may be arranged in a descending order according to the measured concentration required to inhibit tumor cell proliferation by 50% (IC_50_ μ/M). From the results, we can see that compounds 1 and 2 showed significant growth inhibition activity on the 3 tumor cell lines (IC_50_=11.3∼19.8 μ/M), compared to their corresponding organic ligands Py, L_1_ and L_2_ (IC_50_=90.8∼120.5 μ/M).

**Table 2.** Antiproliferative activity IC_50_ (μM) of Py_3_CoL_1_ (1) and Py_3_CoL_2_ (2) and their corresponding organic ligands Py, L_1_ and L_2._

Compounds	IC_50_ (μM)		
	A-431	HT-144	SK-MEL-30
Py	114.5±6.2	120.5±6.1	117.5±4.9
L_1_	93.2±7.1	94.9±6.9	98.8±7.0
L_2_	90.8±3.5	112.6±5.3	111.7±5.9
1	11.3±2.7	17.8±3.1	19.8±4.8
2	16.3±1.8	17.1±2.1	17.4±2.6
Doxorubicin*	0.158±0.067	0.141±0.061	0.180±0.041

Data are reported as means±SE of 3 independent experiments performed in duplicates. IC_50_: Drug concentration required to inhibit tumor cell proliferation by 50% after continuous exposure of 48 h. *Doxorubicin was used as positive control.

In conclusion, we successfully obtained two new Co(II)-coordination compounds (1 and 2) by employing two different flexible Schiff base ligands. From the biological activity investigation, we observed that the antitumor activity of compounds 1 and 2 advanced greatly when organic ligands pyridine and Schiff bases were in coordination with Co ion. However, additional studies are needed to define the mechanism underlying this antitumor activity and evaluate the drug efficacy in *vivo*.
